# Lost time: Perception of events timeline affected by the COVID pandemic

**DOI:** 10.1371/journal.pone.0278250

**Published:** 2023-05-31

**Authors:** Daria A. Pawlak, Arash Sahraie

**Affiliations:** School of Psychology, University of Aberdeen, Aberdeen, United Kingdom; University of Bergamo, ITALY

## Abstract

The need to remember when a past event occurred, is often an everyday necessity. However, placing events in a timeline is seldom accurate and although to some extent modulated by event saliency, on average we are less accurate in remembering a timeline for events happening in the distant past compared to more recent events. 277 participants took part in an online study during May 2022 in which they were asked to state the year in which a number of events took place. The events’ occurrences ranged from 2017 to 2021, with participants choosing one date from the 2016–2022 range. In addition, they completed 4 questionnaires aimed at quantifying their State Boredom; Depression, Anxiety & stress; resilience; and level of activity during the lockdown periods of the COVID pandemic. As expected, the findings showed more errors for distant events than those in 2020, but surprisingly we found a large error for estimating the timing of events that occurred in 2021 matching in the extent to those 3 to 4 years earlier. The findings show that participants were less able to recall the timeline of very recent events coinciding with COVID lockdowns. This increased error in perception of event timeline correlated positively with reported levels of depression & anxiety as well as physical and mental demands during the pandemic, but negatively correlated with measures of resilience. Although measures of boredom showed significant correlations with reported depression & anxiety and physical/mental load, they did not correlate with errors in the perception of the event timeline for 2021. The findings are consistent with poor perception of event timeline reported previously in prison inmates. It is likely that an accurate perception of an event timeline relies on a collection of life events such as birthdays, holidays, travels, etc., anchoring our experiences in the time domain, which was largely absent during COVID restrictions.

## Introduction

The COVID-19 worldwide pandemic began at the end of 2019 as the novel virus spread around the world rapidly. By January 2020, the World Health Organization [1] had declared a public health emergency. In response, many country leaders, imposed lockdown restrictions on their citizens to limit the spread of the virus and allow the health systems to manage the number of hospital admissions [1, 2]. These restrictions included wearing a face covering, keeping a safe distance, or isolating at home and avoiding/minimising social interactions. Although the restrictions were initially expected to last for a brief period, as infection rates increased and new variants of the virus immerged, they were enforced for a significant part of two years.

The main concern throughout the pandemic was to keep people safe, especially the vulnerable individuals which were at greater risk of fatal consequences on their health caused by the virus. Little attention was paid to the consequences that the pandemic related restriction might have on the psychological well-being of individuals. The negative effects of the pandemic on the mental health of individuals and the management of conditions requiring clinical interventions have been significant [3]. The adverse effects of social isolation have led to behavioural effects which are characterised by maladaptive behaviours, defensive responses, and emotional distress, leading to negative emotional experiences including anxiety, fear, boredom, and depression [4, 5].

Time passage in everyday lives fluctuates with an individual’s emotional state, probably as a consequence of changes in the allocation of attention [6]. With increased life uncertainties during the pandemic, individuals may have experienced anxiety-like feelings and negative affects [2], which could lead to a distortion of an individual’s perception of time during the pandemic [7, 8]. Social isolation also increased the feeling of boredom which in turn affects individuals’ relationship with time. With boredom, time begins to appear to be monotonous and extremely long [2, 9]. Furthermore, a study by Micillo and collegues [10] showed that a past negative time perspective, which represents a negative approach to past experiences, was a consistent predictor of anxiety and depression. This shows that individuals negative relationship with the past experiences can negatively influence their mental well-being and thus distort both time perception and emotions. Adams’ Timescape Theory of Time [11, 12] proposes that timescapes are clusters of temporal features which are fluid and inseparable from past experiences. There are three dimensions for timescapes, namely timescale, temporality and fluidity (described below). This theory was adapted by Muth and colleagues [13] to investigate the passing of time in the prison environment. Although drawing a comparison between the prison environment and pandemic related restriction might be seen as an extreme case, we argue that there are similarities in the extent of social isolation in both situations. The governments’ imposed restrictions forced the population to quarantine when infected and minimise social interactions curtailing the daily lives of the population and the subsequent loss of temporal landmarks such as life events contributed to the impairment in time perception [7]. Using the same theoretical framework, we aimed to investigate whether the emotional states of boredom and negative affects experienced during the lockdown period correlated with the ability to correctly report the events’ timeline. Apart from the prison context, the adverse effect of social isolation has also been extensively reported in studies concerning individuals suffering from cancer illness. In an evolutionary concept analysis conducted by Liang and colleagues [14], it was reported that social isolation in cancer patients results in three consequences, low therapeutic compliance, poor health condition and mental health status, and low life quality. Poor mental health and dissatisfaction with life quality due to social isolation creates a risk factor for increased anxiety and depression. The influence of negative affectivity during social isolation is therefore significant and its influence on individuals’ time perception is worth investigating.

In the timescape theory of time, “timescale” is defined by distances and frequencies of repeating events that punctuate one’s life. It helps individuals to make meaning, interpret experiences and create identities [13]. To apply this dimension to the present study a timescale was developed to assess individuals’ ability to judge the timeline of popular events which occurred before, during and after the pandemic related restrictions.

The second time dimension is “temporality” which is used to describe how individuals manage to cope with finitude and finality when they are faced with temporal phenomena of irreversibility and temporariness [13]. The COVID pandemic might be seen as a temporal phenomenon that links to finitude and finality. To measure this dimension, a measure of participants’ coping abilities was included. The Brief Resilient Coping Scale (BRCS) was used to assess participants’ coping abilities [15]. This measure allowed distinguishing the low, medium, and high levels of resilience in individuals during the pandemic.

The last time dimension applied is “time fluidity” which refers to nonlinear, flowing, bidirectional ways in which people experience the past, present, and future. The focus of this dimension is on the quality of the experience of being in time. The lack of time fluidity seems to have a high cost in terms of identity and relationships [13]. Task load can affect time fluidity [5]. The NASA-TXL task load index can be used to assess the average workload of an average day during the period of the pandemic. As shown in the study by Ogden [9], this scale allowed discovering whether the passage of time judgments was affected by the task load that participant had during the pandemic. Similarly, inclusion of the same scale allows investigation of whether the effect of daily load also extends to the perception of event timeline.

In brief, psychological research on short-time perception shows that different emotional states affect the way we perceive the passing of time. Objectively time passes at a constant linear rate. Subjectively, our experience of time is influenced by the activities that we perform and the emotions that we experience [7, 11]. Deprivation of temporal cues affects human ability to situate oneself in the time perspective which in turn influences temporal distortions. In extreme cases of isolation where individuals suffer from complete sensory deprivation, they can become unable to perform correct judgments of time intervals [16]. The pandemic has caused a disconnection between measurable objective time and internally perceived subjective time [9]. It is likely that changed routines and uncertainty about the future contributed to our distorted experience of the passage of time [8, 9, 17]. Thus, we hypothesized that the participants would have the worst memory for more distant past events than for more recent events. Also, those individuals who experienced negative affect and boredom, had low resilience coping abilities, or minimal daily workload during the lock-down period, would perform worse on the judgment of events timeline.

## Materials and methods

### Participants

The sample size was determined based on power analysis conducted on data from a pilot investigation of 105 participants conducted in January 2022, a summary of which can be found on Open Science Forum (https://osf.io/3bnuq). The correlation of error in estimating event timeline for events occurring in 2021 and behavioural measures collected (described below), showed a significant negative correlation with the NASA activity questionnaire (one tail). Power calculations were conducted in G*power to estimate the sample size needed for a significant correlation (two-tailed) at correlation ρ = .174 and α = 0.05, Power (1-β error probability) = .8 with null hypothesis correlation of 0 (lower critical r = -0.123; upper critical r = .1223), led to an estimated sample size of 256 participants. To account for the dropout/incomplete data rate, 278 individuals who did not take part in the pilot study were recruited for the main study. Inspection of data showed that only one participant had to be excluded due to incomplete responses and therefore, all analyses reported here were conducted on a sample of 277 participants (183F, 94M).

Participants were recruited from the participant recruitment platform Prolific (www.prolific.co). Participants had to review and accept the terms of consent form before beginning the study. It was important to ensure that all participants underwent the same periods of restrictions and lockdowns therefore the invitation to participate only extended to those who were resident in the UK for the previous three to four years. We also limited the participation to those 21 years or older to ensure that participants were at least 16 years old when the oldest events took place. Demographical information about participants (age, marital status, employment status) was collected and included in Table 1.

**Table 1:** Sample demographic information frequency and percentages.

	Frequency	percentage
**Age**		
21–29	121	43.7
30–39	113	40.8
40–49	26	9.4
50–59	11	4
60+	6	2.2
**Marital status**		
Single	104	37.5
In partnership	68	24.5
Married	91	32.9
Widowed	1	0.4
Divorced	9	3.2
Prefer not to say	3	1.1
Other	1	0.4
**Employment status**		
Unemployed	18	6.5
Employed full time (home)	107	38.6
Employed full time (work)	47	17
Employed part time (home)	14	5.1
Employed part time (work)	25	9
Student	39	14.1
Retired	3	1.1
Unable to work	8	2.9
Other	16	5.8

The experimental design was a within-subject repeated measure design as all the participants had answered the same questions. The timeline error scores were subject to a one-way ANOVA and correlational investigations were conducted to explore the relationship between timeline errors during the pandemic to other measures of subjective experience.

### Procedure

Ethical approval was granted by the School of Psychology Ethics Board, University of Aberdeen. The study was administered online through the Testable.com platform. The consent sheet was provided online and only participants who expressed their consent by pressing a “I do consent” button were able to continue with the study. Participants on average took under 10 minutes to complete the study online and were awarded £2 for their participation. Data was collected between 4–30 May 2022. Participants first answered some demographic questions before proceeding to complete the following questionnaires. A full list of questions for all questionnaires outlined below is included in the S1 File (see S1 File for Measurement Batteries).

### The Multi-Dimensional State Boredom Scale (MSBS)

MSBS [18] is composed of 29-items that aim to measure the state boredom. The scale was adjusted to cover the period of the COVID-19 pandemic by having the “During the period of the pandemic…” phrase added before every question (example of item: During the period of pandemic, time dragged on). Participants were able to supply their answers based on a seven-point Likert scale from 1 (strongly disagree) to 7 (strongly agree). Although the questionnaire includes five subscales, for the purpose of our research the general state of boredom was calculated by summing the scores of all items. Scores range from 0 to 203, with higher scores indicating higher boredom levels in individuals.

### The Depression, Anxiety, Stress Scale-21 (DASS-21)

The DASS-21 [19] is a short version of the DASS-42 and consists of 21-items that measure internalised distress. This scale was adjusted to measure the period of the pandemic by having the “During the period of the pandemic…” phrase added before every question. (Item example: During the pandemic, I had nothing to look forward to). Participants were asked to answer by indicating the severity with which each item reflected participants’ experiences, from the range, 0 (Did not apply to me) to 3 (Applied to me very much or most of the time). Response scores from DASS-21 should be doubled to enable the classification of each dimension (depression, anxiety, stress) against specified cut-offs. Doubling the scores reflects equivalent scores to the full DASS-42 version [20].

### The Brief Resilient Coping Scale (BRCS)

The BRCS [21] aims to capture individual tendencies to deal with stress by discovering and effectively using coping strategies which allow individuals to actively solve problems under stressful circumstances. To measure the participants’ resilience during the lockdown period, the instructions of BRCS were also adjusted, and they were asked to consider how well the statements within the scale described their behaviour and actions during the pandemic. The scale includes four statements that can be answered on a 5-point scale, from 1 (does not describe me at all) to 5 (describes me very well). A score within the three ranges of 4–13; 14–16; and 17–20 indicates low, medium and high resilient copers respectively.

### National Aeronautics and Space Administration-Task Load Index (NASA-TLX)

NASA-TLX [22] aims to measure the subjective daily workload of participants by asking 6 questions about mental demand, physical demand, temporal demand, performance, effort and frustration. Here, the instructions and the questions were adjusted to measure the period of lockdown. Participants were asked to take themselves back to the time of lockdown and try to rate each of the 6 items based on their feelings at that time (item example: How mentally demanding were your daily tasks during lockdown?). Answers were obtained based on a 5-point Likert scale, from 1 (very low) to 5 (very high). High scores indicated greater task demand.

### Event timeline measure

A timescale was developed to assess objective time perception. It included 20 events, with 5 events per year between 2017 and 2021 and events were highly publicised in the media. Participants could choose to respond by a year in the range of 2016–2022 to avoid tactical answers. As all participants were UK residents over the past 4 years. Some chosen events were highly related to the UK such as “In which year Brexit was finalised?” or “When did Meghan Markle join the British Royal Family?”. Some global events were also included such as “When did the COVID-19 vaccination program begin?” or “Evergreen container ship got stuck in the Suez Canal”. A full list of events and the dates in which they occurred are included in the S1 File.

Data was downloaded from Testable.com platform and screened for completeness. Individual scores from NASA-TLX were averaged, while scores from MSBD, DASS-21 and BRCS were summed up. Descriptive statistics, frequencies, and percentages were calculated for the demographical data. For each participant, the error in the event timeline was calculated by subtracting the reported year of the event from the actual event year and then averaging for all events in that year.

## Results

### Event timeline

**Fig 1:**
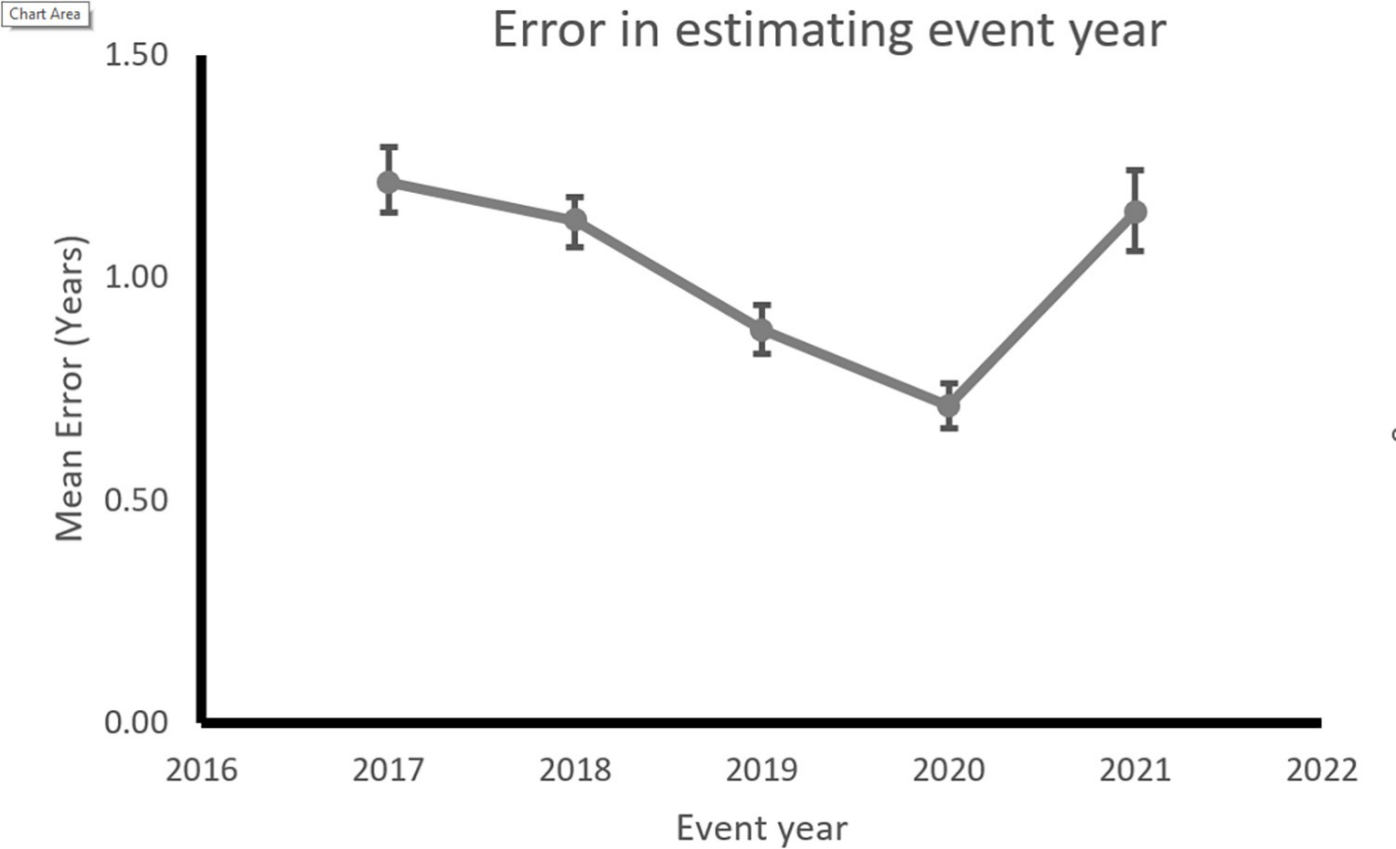
Mean Error in estimating the event year for a 5 year period. Error bars indicate 95% Confidence Interval of the mean.

The plot of mean error in estimating the event timeline is plotted against the event year in Fig 1 (see S1 File for all descriptive statistics). As predicted, there is more uncertainty in guessing the event year for more distant events. The error in event time was the highest in 2017 (M = 1.21, SD = 0.58) and reduced for the subsequent years 2018 (M = 1.13, SD = 0.47), 2019 (M = 0.88, SD = 0.46) and 2020 (M = 0.71, SD = 0.44). Surprisingly this measure increased in 2021 (M = 1.15, SD = 0.73). Analysis of variance showed a main effect of Event Year on error estimate, *F*(4,276) = 44.83, *p* < .001. Indeed post-hoc analysis (Bonferroni corrected) showed that the error in estimating event timeline in 2021 was significantly higher than those for 2020 (t(276) = 8.42, p < .001, Cohen’s d = 0.506); and 2019 (t(276) = 5.353, p < .001, Cohen’s d = 0.322) but not significantly different than 2018 and 2017 (Pbonf = 1).

### Correlational analysis

**Fig 2:**
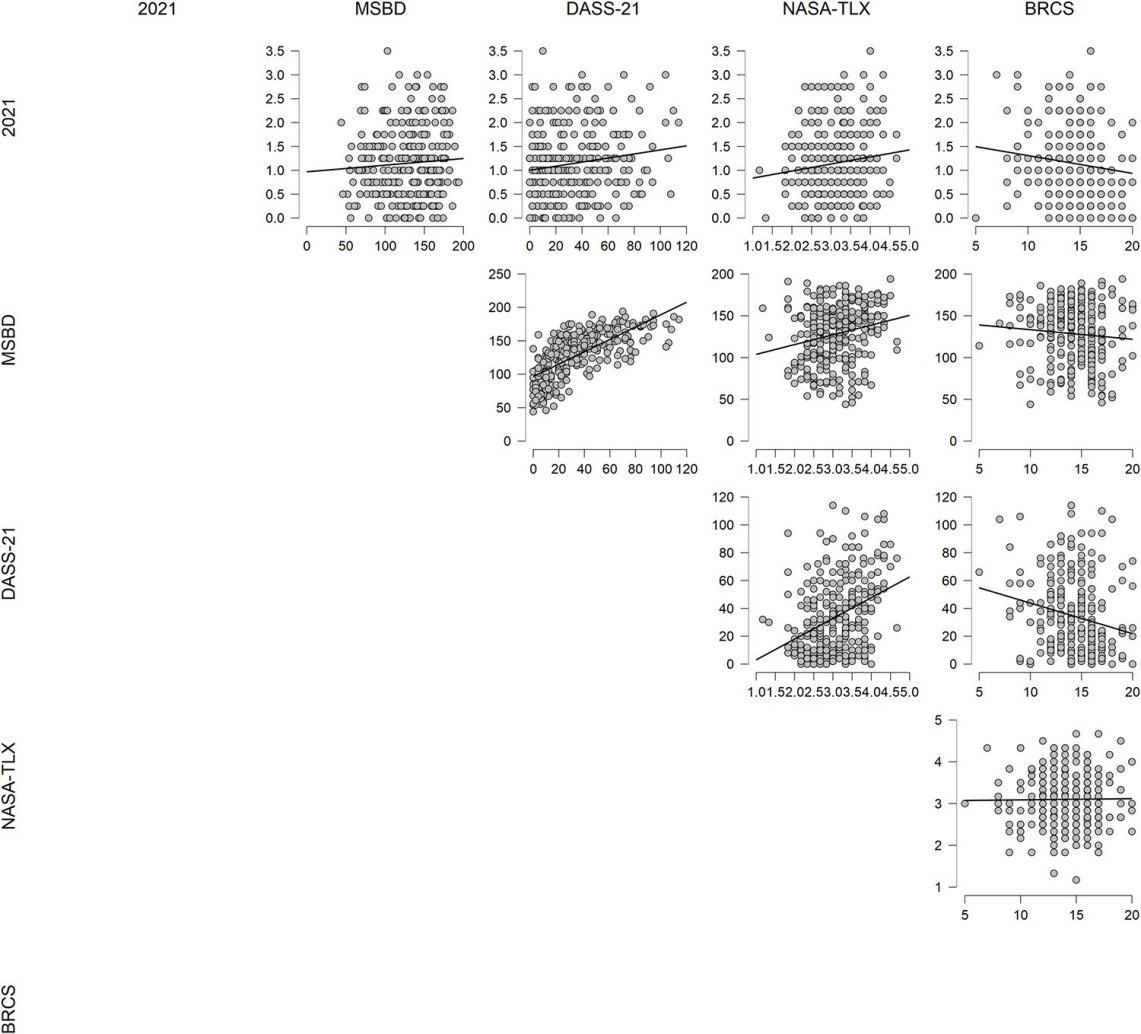
Correlation plots for estimated error in remembering events that took place in 2021 versus four behavioural measures MSBD, DASS-21, NASA-TLX and BRCS.

The finding that the error in estimating the event timeline for 2021 was significantly worse than the two years prior and the magnitude was similar to those in 2017 and 2018 was surprising. Therefore, further correlational analysis was conducted to find out if the deteriorated performance for remembering the events in 2021 correlated with performance in the other 4 measures included, MSBD, DASS-21, NASA-TLX and BRCS (see S1 File for all descriptive statistics). This correlation matrix is shown in Table 2, and correlation plots are shown in Fig 2.

**Table 2:** Pearson correlations.

			Pearson’s r	p
2021	-	MSBD	0.067	0.265
2021	-	DASS-21	0.156	0.009
2021	-	NASA-TLX	0.131	0.030
2021	-	BRCS	-0.129	0.031
MSBD	-	DASS-21	0.703	< .001
MSBD	-	NASA-TLX	0.214	< .001
MSBD	-	BRCS	-0.082	0.173
DASS-21	-	NASA-TLX	0.359	< .001
DASS-21	-	BRCS	-0.208	< .001
NASA-TLX	-	BRCS	0.011	0.853

The estimated error for remembering events in 2021 correlated positively with scores in both Depression, Anxiety, Stress Scale -21 (DASS-21) and NASA -Task Load Index, so those with higher levels of internalised stress, similar to those with mentally and physically more demanding jobs could not accurately remember the timing of events that occurred in 2021. However, mean errors in estimating the event years correlated negatively with scores on Brief Resilient Coping Scale (BRCS), that is, those with higher indicators of resilience were better at remembering the timing of events that happened in 2021. Surprising there was no significant correlation with the scores on the Multi-Dimensional State Boredom Scale (MSBS), although MSBS correlated significantly with both DASS-21 and NASA-TLX; with those with higher levels of depression and anxiety as well as those under both physical and mental strain reported higher levels of stress. The negative correlation between boredom and resilience was in the right expected direction but not significant. The findings also show that the measure of state anxiety and depression correlated positively with reports of being under physical and mental strain, but negatively with resilience. It is of note that although correlations between anxiety and measures of boredom; or load/stress are higher, the correlations with estimation of event timeline although significant, are generally lower.

## Discussion

This study was conducted to test observations made by a number of clinician colleagues who routinely take symptoms and history from patients. There is a high demand for access to healthcare following the pandemic, but a common observation has been that patients cannot give an accurate timeline, including the start and progression of their symptoms with errors of one to two years being common. Should these observations be an adverse effect of the pandemic restrictions on social activities, we expected them to apply to the wider populations and extend beyond matters related to an individual’s health. Hence, we developed a questionnaire to measure the accuracy of placing popular events in a timeline. Distortion of time perception as a consequence of psychological states has been well established. These include the effect of emotion and arousal in time perception for short durations (minutes/hours) during life-threatening accidents [22] or high-adrenaline sports such as skydiving [23], to the perception of passing years in prison inmates [24]. The adverse long-term effects of isolation have been reported in several studies and commonly show an increase in the experienced negative affectivity by individuals. The emotion-induced distortions have been explained to be influenced by the underlying loneliness that participants have reported during social isolation periods [8, 25]. The feeling of loneliness leads to emotional distortions such as higher levels of stress, anxiety, and depression which were further found to adversely influence the time judgment abilities [8, 25]. Combining this knowledge with the present research provides concrete evidence of how the imposed lockdown distorted not only the emotional stability but also the ability to judge temporal landmarks. For the events that occurred early or later in the year, misplacing the event by a short period (e.g., 2 months) could potentially lead to errors in measurements. Analysis reported in the S1 File show that the main findings reported here are robust and maintained even if every participant made the same error.

To study time perception in the context of psychological states, we administered four instruments to place the findings in the context of boredom, depression anxiety and stress as well as mental and physical demand experienced during the pandemic. Whilst the pandemic and associated restrictions and uncertainties induced negative affect [26], an individual’s level of resilience can also modulate their behaviour and perception [27]. Therefore, a brief resilience questionnaire was also included.

Here we have reported evidence that similar to other adverse effects of enforced isolation, the perception of psychological time was also deteriorated. This finding is in line with Chaumon and colleagues [25] that reported lengthening of the temporal distances. Although their study did not measure event perception, but rather clock duration in either case a retrospective underestimation of the elapsed time was found. Therefore, the distortion of time phenomenon seems to be persistent in both long- and short-time judgments. The errors in estimating the event timeline were surprisingly high for the more recent events and correlated positively with both depression, anxiety, stress scale and physical and mental load, but negatively correlated with the measure of an individual’s level of coping and resilience. It was surprising to find no significant correlation with boredom. However, this lack of correlation is likely due to boredom itself not being diagnostic, but a product of a wide variety of factors. We have found that boredom correlated with both measures of DASS-21 and NASA-TLX, yet not significantly with resilience. Finally similar to other studies we show that depression and anxiety are positively correlated with internal and external pressures, and inversely with coping and resilience [8, 28]. This is in agreement with a systematic review and meta-analysis produced by Salari and colleagues [5] who analysed 17 studies on the prevalence of stress, anxiety, and depression in the general population in the first year of the pandemic. Their results show that the pandemic increased anxiety in individuals which further influenced a surge in mental health disorders across the globe.

Within the theoretical framework, a Timescape refers to how an individual perceives the subjective passage of time. From the timescape perspective, the linear passage of physical time is transformed within the framework of the social perception of time [11]. Our interactions with the environment, for example, affect the temporality time dimension. Therefore, how an individual interacts with complex environmental stresses, can influence the way they place events in the timescape. On a related note, pandemic restrictions started in 2020, but we found the maximum distortion of timeline for 2021. Covid restrictions were non-existent in the first quarter of the year and the associated restrictions, public briefings and societal changes were certainly novel and unprecedented for many of the subsequent months. All these highly unusual events themselves can form anchor points in timescape and therefore leading to more accurate estimations of event timelines. We would argue that the novelties had been reduced by 2021 where other effect of social isolations also became more pronounced including the spikes in mental health deteriorations. Some studies emphasize the importance of having strong resilience coping strategies in place to be utilized in stressful situations to restrict adverse effects [15, 29]. By using BRCS to measure resilience we have obtained a subjective measure of how an individual responded to life stresses. Our findings demonstrate that individuals in this study were on average moderate resilient copers. It is likely that finding effective coping strategies was challenging for most under the stressful temporal phenomenon of COVID-19. Hence, the weak resilient copers were found to perform worse on the timescale task. Here we also show that the perceived load and probably stresses associated with it as measured using NASA-TLX [30] can affect the accuracy of estimating the event timeline. This extends the previous reports that stresses can alter the perception of the passage of time itself [9] such as a perceived “slowing down” of time due to reduced task load.

As noted earlier, the current investigation was inspired by observations made by our clinical colleagues that post-pandemic, patients are unable to give an accurate timeline of their ailments. This anecdotal evidence pointed towards an impairment of autobiographical memory for events happening during the pandemic in general population who are accessing clinical services. Systematically assessing changes in autobiographical memories in a large sample of population has not been feasible. We have therefore examined the broader question of a general deterioration in event timeline. Our study has clearly demonstrated that these deficits exist for societal based events (non-autobiographical) that occurred during the pandemic. The correlational study is limited in determining the causal roots of the deficit, nevertheless the findings are a pointer on the probable factors that can influence the extent of the deficits.

In conclusion, although the direct adverse effect on the health of a population by a pathogen can be well documented and the optics of disease such as reported hospital admissions can largely underpin the management decisions in a pandemic, the resultant interventions can also have dramatic hidden adverse effect on a variety of cognitive processes. Here we have demonstrated such influences on the perception of event timelines in individuals.

## Supporting information

S1 File List of events and dates, descriptive statistics and measurement batteries. (DOCX)
