# Supplementary material for: Lost time: Perception of events timeline affected by the COVID pandemic
Source: PLoS One. 2023 May 31;18(5):e0278250. doi: 10.1371/journal.pone.0278250 (PMC10231762; doi:10.1371/journal.pone.0278250)
Supplement: S1 File — (DOCX) [file pone.0278250.s001.docx]

**Supplementary material**

**Event Timeline measure**

Instructions:

Try to think about the below-mentioned events and make a guess about when they have happened. No cheating please!

- - - 1. Poisoning of the Russian double-agent Sergei Skripal and his daughter in Salisbury
      2. Prince Harry and Meghan Markle's interview with Oprah Winfrey
      3. Data mining scandal involving Facebook and Cambridge Analytica
      4. Terrorist attack at Palace of Westminster in London
      5. Fire devastated Paris historic Notre Dame Cathedral
      6. Space tourism began with the first test flight taken to the galaxy
      7. Donald Trump was impeached
      8. The first occurrence of the coronavirus disease
      9. Grenfell Tower fire
      10. The killing of George Floyd – beginning of Black Lives Matter movement
      11. Destructive fires in the Amazon
      12. Terrorist attack at the Manchester Arena during Ariana Grande concert
      13. Evergreen container ship got stuck in the Suez Canal
      14. Stephen Hawking passed away
      15. Donald Trump was banned on Facebook
      16. Boris Johnson became a prime minister
      17. Brexit was finalized
      18. Meghan Markle joined the British royal family
      19. Beginning of the vaccination program against coronavirus disease
      20. Worldwide women march held to support gender equality, civil rights, and other issues that were expected to face challenges under newly inaugurated U.S. Pres. As well as, the beginning of the #metoo movement.

Participants had the option to select the presumed date between year 2016 and 2022.

Dates for the events above:

1. 03/2018
2. 03/2021
3. 03/2018
4. 03/2017
5. 04/2019
6. 05/2021
7. 02/2020
8. 12/2019
9. 06/2017
10. 05/2020
11. 01/2019
12. 05/2017
13. 03/2021
14. 03/2018
15. 06/2021
16. 07/2019
17. 01/2020
18. 05/2018
19. 12/2020
20. 01/2017

Descriptives for dates and parameters measure

| **Descriptive Statistics** | | | | | | | | | | | | | | | | | | | |
| --- | --- | --- | --- | --- | --- | --- | --- | --- | --- | --- | --- | --- | --- | --- | --- | --- | --- | --- | --- |
|  | | **2017** | | **2018** | | **2019** | | **2020** | | **2021** | | **MSBD** | | **DASS** | | **BRS** | | **NASA** | |
| **Mean** |  | 1.214 |  | 1.128 |  | 0.8827 |  | 0.7121 |  | 1.147 |  | 128.3 |  | 34.30 |  | 14.33 |  | 3.101 |  |
| **Std. Deviation** |  | 0.5758 |  | 0.4680 |  | 0.4582 |  | 0.4381 |  | 0.7272 |  | 35.11 |  | 26.63 |  | 2.520 |  | 0.6429 |  |
|  | | | | | | | | | | | | | | | | | | | |

Fig S1: The violin plots depicting the distribution of scores for each of the four questionnaires MSBD, DASS, BRCS and NASA administered in the study.


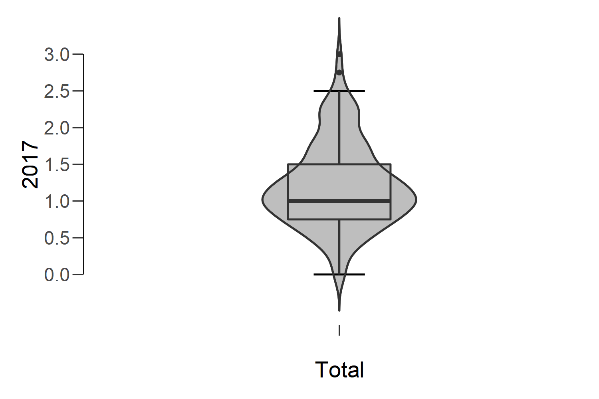

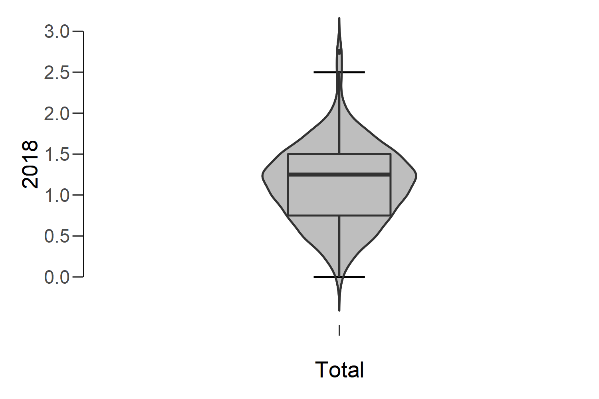

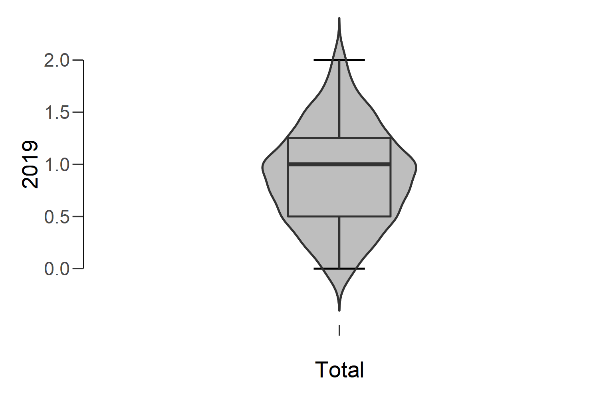

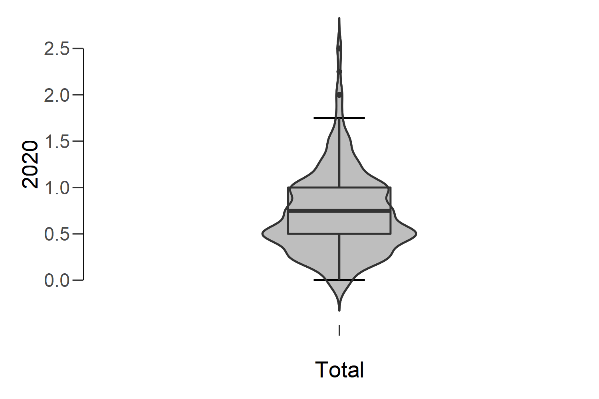

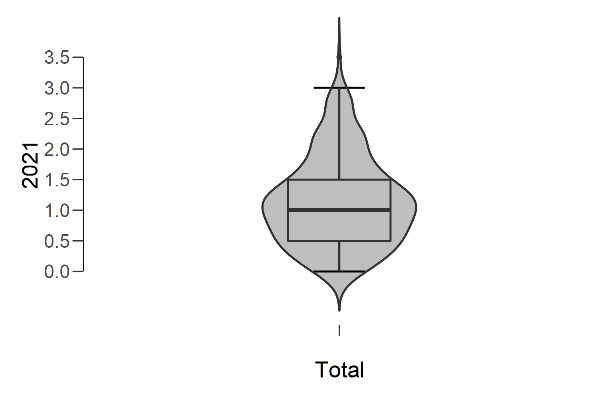

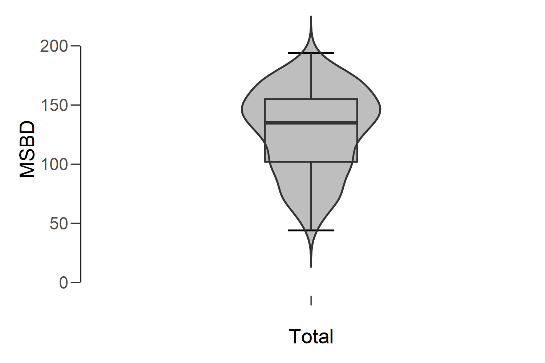

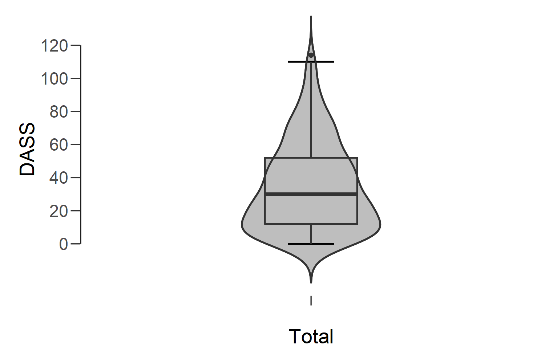

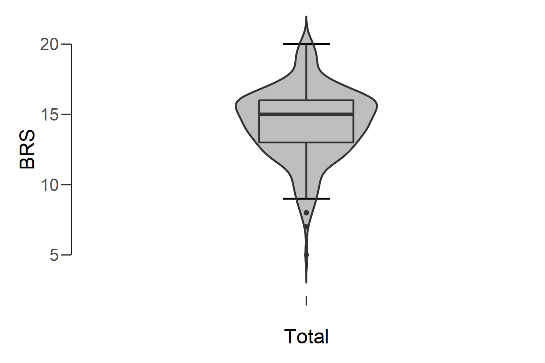

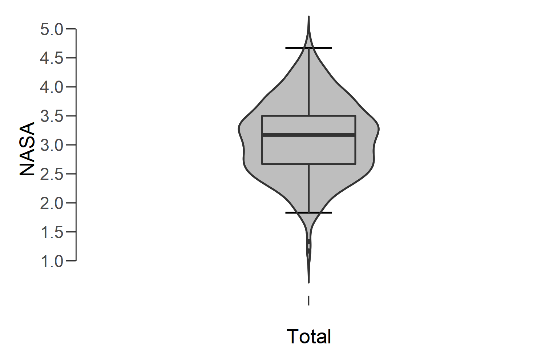


**Effect of restricting analysis to year of event timeline**

A list of events and the year that they occurred are provided above. In the analysis reported in the manuscript, we limited responses to a given year, rather than using a shorter timeframe such as a month in a year. As events can happen anytime in a year, we have conducted a reality check to see what the effect on the findings would be if the participants made an error and attributed an event to happening either two months earlier or later. For events happening in the last or the first two months of the year, this would lead to change in error score by one year for the given event. For our data it would potentially affect 6 questions. We found 6 questions (1 in 2017; 2 in 2019; 3 for 2020) that would fall in this category. We have gone through each participants answer and modelled the data to result in minimum error. (E.g., for an event in January or February 2019, if the answer was 2018, we changed it to 2019 to reduce error to zero instead of 1 for that participant, in that question). We then recalculated all means and t-tests for these.

Figure S2: Plot of mean error for the event timeline reported in the manuscript and the modelled data assuming that every participant made an error for events taking place in the first or last 2 months of the year.

Above shows that there is a reduction on the size of the error for the three years (2017, 2019&2020). Crucially, we run the pairwise comparisons for 2021 against other years and show that all significant findings, still hold as shown below (the modelled numbers are denoted by affix A). Therefore, our conclusions are not affected by this possibility.

| **Paired Samples T-Test** | | | | | | | | | | | | | | | | | |
| --- | --- | --- | --- | --- | --- | --- | --- | --- | --- | --- | --- | --- | --- | --- | --- | --- | --- |
|  | | | | | | | | | | | | | | **95% CI for Cohen's d** | | | |
|  | |  | |  | | **t** | | **df** | | **p** | | **Cohen's d** | | **Lower** | | **Upper** | |
| 2021 |  | - |  | 2020 |  | 8.422 |  | 276 |  | < .001 |  | 0.506 |  | 0.381 |  | 0.631 |  |
| 2021 |  | - |  | 2019 |  | 5.353 |  | 276 |  | < .001 |  | 0.322 |  | 0.201 |  | 0.442 |  |
| 2021 |  | - |  | 2018 |  | 0.384 |  | 276 |  | 0.701 |  | 0.023 |  | -0.095 |  | 0.141 |  |
| 2021 |  | - |  | 2017 |  | -1.200 |  | 276 |  | 0.231 |  | -0.072 |  | -0.190 |  | 0.046 |  |
| 2020 |  | - |  | 2019 |  | -5.021 |  | 276 |  | < .001 |  | -0.302 |  | -0.422 |  | -0.181 |  |
| 2021A |  | - |  | 2020A |  | 12.671 |  | 276 |  | < .001 |  | 0.761 |  | 0.627 |  | 0.895 |  |
| 2021A |  | - |  | 2019A |  | 7.131 |  | 276 |  | < .001 |  | 0.428 |  | 0.305 |  | 0.551 |  |
| 2021A |  | - |  | 2018A |  | 0.384 |  | 276 |  | 0.701 |  | 0.023 |  | -0.095 |  | 0.141 |  |
| 2021A |  | - |  | 2017A |  | -0.353 |  | 276 |  | 0.724 |  | -0.021 |  | -0.139 |  | 0.097 |  |
| 2020A |  | - |  | 2019A |  | -8.612 |  | 276 |  | < .001 |  | -0.517 |  | -0.642 |  | -0.392 |  |
|  | | | | | | | | | | | | | | | | | |

Admittedly we did not have much choice is setting up a higher limit than 2022, but the choice of 2016 limits the extent of the error possible for distant past events. Looking at the graph above, the data seems to asymptotes for 2017 and it is probable that a wider range would result in higher error (i.e., the lines looking more linear than asymptotic). However, we also wanted to avoid the criticism that the lower errors for the recent events compared to the higher errors for past events were an artifact of having a smaller range of possibilities for recent events and higher one for the past events! Therefore, it is a part of trade-off that one has to make in study design. Having the limitations of the study in mind, we have deliberately not expanded on the shape and properties of the error for time estimate for 2017 and earlier years.

**The Multi-Dimensional State Boredom Scale (MSBS)**

Instructions.

Please respond to each question indicating how you felt during the pandemic, about yourself and your life, even if it is different from how you usually feel. Use the following choices: 1 = Strongly disagree; 2 = Disagree; 3 = Somewhat disagree; 4 = Neutral; 5 = Somewhat agree; 6 = Agree; and 7 = Strongly agree.

If you are uncomfortable with any of the questions, remember that you can skip it or withdraw from the study at any point.

1. During the period of the pandemic, time was passing by slower than usual.
2. During the period of the pandemic, I was stuck in a situation that I felt was irrelevant.
3. During the period of the pandemic, I was easily distracted.
4. During the period of the pandemic, I was lonely.
5. During the period of the pandemic, everything seemed to be irritating me.
6. During the period of the pandemic, I wished time would go by faster.
7. During the period of the pandemic, Everything seemed repetitive and routine to me.
8. During the period of the pandemic, I felt down.
9. During the period of the pandemic, I was forced to do things that have no value to me.
10. During the period of the pandemic, I felt bored.
11. During the period of the pandemic, Time dragged on.
12. During the period of the pandemic, I was more moody than usual.
13. During the period of the pandemic, I was indecisive or unsure of what to do next.
14. During the period of the pandemic, I felt agitated.
15. During the period of the pandemic, I felt empty.
16. During the period of the pandemic, It was difficult to focus my attention.
17. During the period of the pandemic, I wanted to do something fun, but nothing appealed to me.
18. During the period of the pandemic, Time was moving very slowly.
19. During the period of the pandemic, I wished I was doing something more exciting.
20. During the period of the pandemic, My attention span was shorter than usual.
21. During the period of the pandemic, I was impatient.
22. During the period of the pandemic, I wasted time that would be better spent on something else.
23. During the period of the pandemic, My mind was wandering.
24. During the period of the pandemic, I wanted something to happen but I was not sure what.
25. During the period of the pandemic, I felt cut off from the rest of the world.
26. During the period of the pandemic, it seemed like time was passing slowly.
27. During the period of the pandemic, I was annoyed with the people around me.
28. During the period of the pandemic, I felt like I’m sitting around waiting for something to happen.
29. During the period of the pandemic, It seemed like there’s no one around for me to talk to

**The Depression, Anxiety, Stress Scale -21 (DASS-21)**

Instructions:

Please think back to the time when we were in lockdown and try answering the questions below referring to your felt emotions at that time. Please use the following rating: 0- did not apply to me, 1- applied to me to some degree/some of the time, 2- applied to me to a considerable degree or a good part, 3- applied to me very much or most of the time.

If you are uncomfortable with any of the questions, remember that you can skip it or withdraw from the study at any point.

1. I found it hard to wind down
2. I was aware of dryness of my mouth
3. I couldn’t seem to experience any positive feeling at all
4. I experienced breathing difficulty (e.g. excessively rapid breathing, breathlessness in the absence of physical exertion)
5. I found it difficult to work up the initiative to do things
6. I tended to over-react to situations
7. I experienced trembling (e.g. in the hands)
8. I felt that I was using a lot of nervous energy
9. I was worried about situations in which I might panic and make a fool of myself
10. I felt that I had nothing to look forward to
11. I found myself getting agitated
12. I found it difficult to relax
13. I felt down-hearted and blue
14. I was intolerant of anything that kept me from getting on with what I was doing
15. I felt I was close to panic
16. I was unable to become enthusiastic about anything
17. I felt I wasn’t worth much as a person
18. I felt that I was rather touchy
19. I was aware of the action of my heart in the absence of physical exertion (e.g. sense of heart rate increase, heart missing a beat)
20. I felt scared without any good reason
21. I felt that life was meaningless

**The Brief Resilient Coping Scale (BRCS)**

Instructions:

Consider how well the following statements describe your behavior and actions during the pandemic. Please use the following rating: 1- does not describe me at all, 2- does not describe me, 3- neutral, 4- describes me, 5- describes me well

If you are uncomfortable with any of the questions, remember that you can skip it or withdraw from the study at any point.

- 1. I look for creative ways to alter difficult situations.
  2. Regardless of what happens to me, I believe I can control my reaction to it.
  3. I believe I can grow in positive ways by dealing with difficult situations.
  4. I actively look for ways to replace the losses I encounter in life.

**National Aeronautics and Space Administration-Task Load Index (NASA-TLX)**

Instructions:

Please take yourself back to the time of lockdown and try to answer those questions based on your feelings then. Please use the following rating: 1- very low, 2- low, 3- neutral, 4- high, 5- very high

If you are uncomfortable with any of the questions, remember that you can skip it or withdraw from the study at any point.

1. Mental demand
   1. How mentally demanding were your daily tasks during lockdown?
2. Physical demand
   1. How physically demanding were your daily tasks during lockdown?
3. Temporal demand
   1. How hurried or rushed was the pace of the tasks awaiting completion during lockdown?
4. Performance
   1. How successful were you in accomplishing what you were asked to do?
5. Effort
   1. How hard did you have to work to accomplish your level of performance?
6. Frustration
   1. How insecure, discouraged, irritated, stressed and annoyed were you?
